# Integration of pharmacodynamics, network pharmacology and metabolomics to elucidate the effect and mechanism of Jingfang Granule in the treatment of Paraquat induced Pulmonary fibrosis

**DOI:** 10.1371/journal.pone.0318246

**Published:** 2025-02-18

**Authors:** Rujing Yue, Tianye Yang, Dejun Niu, Zhen Zeng, Xishuang Wang, Lihong Pan, Jingchun Yao

**Affiliations:** 1 State Key Laboratory of Integration and Innovation of Classic Formula and Modern Chinese Medicine, Lunan Pharmaceutical Group Co. Ltd, Linyi, China; 2 Department of Medicine and Pharmacy, Wuhan University, Wuhan, Hubei, China; Shandong Cancer Hospital and Institute Shandong First Medical University and Shandong Academy of Medical Sciences: Shandong Cancer Hospital and Institute, CHINA

## Abstract

**Objective:**

One of the main risk factors of COVID-19 is Pulmonary fibrosis (PF). The protective effect of Jingfang Granule (JF) to bleomycin-induced PF has been confirmed in our previous studies. This work was designed to reveal the effect and mechanism of JF on PF which induced by Paraquat (PQ).

**Methods:**

In this study, the PF mice model was induced by PQ with the administration of 1, 0.5, and 0.25 g/kg JF or Nintedanib (NTNB) 45 mg/kg by oral administration. The ameliorating effects of JF were reflected by the survival curve and lung coefficient. And the pathological alterations of lung were observed by H&E, Masson and Sirius red staining. Then, the expression of fibrosis-associated protein α-SMA and TGFβ1/Smad2,3 signaling pathway was detected by immunohistochemistry and western blot. An integrated approach combined metabolomics with network pharmacology was applied to recognize the mechanism of JF on ameliorated the PQ-induced PF, and the result of integrated was verified by western blot.

**Results:**

The experiment results showed that JF could inhibit the progression of PQ-induced PF and delay the death of mice after PQ poisoning, and the inhibit effect was similar to NTNB. JF also reduced fibroblasts in lung tissue of the PF mice model by significantly down- regulated the expression of α-SMA and TGFβ1/Smad2,3 signaling pathway. In addition, JF intervened 16 serum metabolites compared with PQ-induced PF mice, and the differential metabolites were linked 241 corresponding targeted proteins obtained by database, which have 79 common targets to JF related targets. The integrated results of metabolomics, network pharmacology and western blot showed that apoptosis was a crucial way for JF to relieve the PQ-induced PF, and JF regulated the signals of Bcl-2, Bax, Caspase-3 protein and PI3k/Akt pathway to inhibit the apoptosis.

**Conclusion:**

These findings demonstrate that JF down-regulated the TGFβ1/Smad2,3 signaling pathway to reduce the fibroblasts, regulate the expression of Bcl-2, Bax, Caspase-3 and PI3k/Akt pathway to inhibit the apoptosis, and display a favorable effect on inhibiting the development of pulmonary fibrosis and delaying the death of PQ-induced PF mice.

## 1. Introduction

The pandemic COVID-19 has plagued the world for 4 years, although the outbreak has been largely contained, its after-effects are still persisting. Pulmonary fibrosis (PF) is a key feature of COVID-19, the rate of COVID-induced PF may exceed 30% according to Vasarmidi [1] and Rai [2]. Paraquat (PQ) is a kind of toxic bipyridilium pesticides, which was widely used for weeding. It is lethal even in small amounts (15–20 mL of 20% w/v) [3]. Victim usually succumbs to respiratory failure as a result of progressive PF [4]. Regrettably, there is no available antidote specifically for PF, it would be significant to find treatments or interventions to reduce the mortality of PF.

Nowadays, traditional Chinese medicine (TCM) is widely accepted in clinic, because of its characteristics of multi-component and multi-target with few side effects. Jingfang granula (JF) was a modern formulation derived from Jingfang Baidu Powder, which was recorded in *She-Sheng-Zhong-Miao-Fang* manuscript, a book of traditional Chinese medicine prescription from Ming dynasty. It is composed of eleven herbal medicines: Jingjie (*Schizonepeta tenuifolia* (*Benth.*) *Briq*), Fangfeng (*Saposhnikovia divaricate* (*Turcz.*) *Schischk*), Qianghuo (*Notopterygium incisum*), Chaihu (*Bupleurum chinense* DC), Qianhu (*Peucedanum praeruptorum* Dunn), Chuanxiong (*Ligusticum striatum* DC), Zhiqiao (*Aurantii Fructus* L), Fuling (Poria cocos (Schw.) Wolf), Jiegeng (*Platycodon grandiflorus* (*Jacq.*) A.DC), and Gancao (*Glycyrrhiza uralensis Fisch. Ex* DC), at a ratio of 3:3:3:3:3:3:3:3:3:3:1, respectively (dry weight). JF is practicable for the beginning of the plague, and it is used clinically for the treatment of epidemic and infectious diseases, such as acute viral upper respiratory tract infection, dengue fever, influenza A (H1N1), chickenpox, and mumps [5]. In previous studies, JF has shown obvious therapeutic effect on CCl_4_-induced liver fibrosis, and its mechanism may be related to reducing the expression of pro-inflammatory factors, anti-oxidation, and regulating TGF-β/Smad4 signaling pathway [6]. It is reported that JF also can alleviate bleomycin-induced acute lung injury via CD200-CD200R immunoregulatory pathway [7]. Prim-O-glucosylcimifugin, neohesperidin, and naringin as the major compounds from JF could inhibit severe acute respiratory syndrome coronavirus 2 (SARS-CoV-2) virus proteases 3CL^pro^ and PL^pro^ [8], and JF is recommended by the National Health Commission of the People’s Republic of China to treat 2019-novel coronavirus-related pneumonia.

The role of JF on PQ-induced PF and its underlying mechanisms remain to be explored. Metabolomic was employed in this study to understand the performance of metabolites, identifying, quantifying them, and thus understanding its mechanism of action [9]. In addition, network pharmacology is another widely chosen tool to predict the pharmacodynamic material basis of herbs and their mechanism [10]. It was used to enrich the targets of metabolic differentials obtained from metabolomics analysis, map them with the targets of JF, and perform the GO and KEGG pathway enrichment analysis.

Therefore, based on the therapeutic effect of JF on injury of lung and the influenza, we wondered whether JF play regulatory roles in the occurrence and development of PF. The study integrates the pharmacodynamics, network pharmacology, and metabolomics, and aims to evaluate the therapeutical effect of JF on PQ induced-PF and explore the underlying mechanisms which JF relieves the PF.

## 2. Materials and methods

### 2.1. Reagents and antibodies

JF was supplied by Lunan Hope Pharmaceutical Co. Ltd. (Linyi, China). Paraquat (PQ) was purchased from the Meryer Biochemical Technology (Shanghai, China). Nintedanib (NTNB) was purchased from Macklin Biochemical Technology Co., Ltd. (Shanghai, China). Hematoxylin-Eosin (H&E) Staining Kit and Modified Masson’s Trichrome Stain Kit were purchased from Solarbio (Beijing, China). Antibodies used for Western blot included α-SMA (Cat#19245S), Caspase-3 (Cat#9661S), Smad2, 3 (Cat#8685S), p-Smad2, 3 (Cat#8828S), Erk (Cat#9102S), p-Erk (Cat#4376S), PI3k (Cat#4292S), p-PI3k (Cat#17366S), Akt (Cat#9271S), p-Akt (Cat#4060S), Bax (Cat#14796S), α-Tubulin (Cat#2144S) (Cell Signaling Technology, Boston, USA); TGF-β1 (Cat#ab215715), Bcl-2 (Cat#ab182858) (Abcam, UK), all antibodies were used at 1:1000 dilution. BeyoECL Plus and Radio Immunoprecipitation Assay (RIPA) lysis buffer were purchased from Beyotime technology (Shanghai, China).

### 2.2. Pulmonary fibrosis and treatment of animals

ICR male mice aged 4 ~ 6 weeks were purchased from Beijing Vital River Laboratory Animal Technology (certificate No.: SCXK (Jing) 2021-0006, Beijing, China). Mice were placed in the environment of 22-24°C and the constant humidity (50% ±  1), and had common feedstuff and drank water freely. All experimental procedures were in accordance with “Guidelines and Use of Laboratory Animals”, and approved by the Experimental Animal Ethics Committee at State Key Laboratory of Integration and Innovation of Classic Formula and Modern Chinese Medicine (Animal Ethics Approval Number: AN-IACUC-2022- 113) to minimize the suffering of animals as much as possible.

All mice were divided into six groups (n = 20) at random: Control, PQ (20 mg/kg), PQ (20 mg/kg) + JFH (1.0 g/kg), PQ (20 mg/kg) + JFM (0.5 g/kg), PQ (20 mg/kg) + JFL (0.25 g/kg) group, and PQ (20 mg/kg) + NTNB (45 mg/kg). All mice but normal group were administered with 20 mg/kg PQ every day via intragastric administration [11] to acquire the PQ-induced PF, mice in control group were given normal saline. All of group were administered for 14 days.

### 2.3. Sample collection and preparation

At the end of experiment, mice were narcotized with 1% pentobarbital solution, blood samples were taken from the abdominal aorta and centrifuged at 3000 rpm/min for 10 min at 4°C.

The lung tissues were removed and divided, the right lower lobe of the lung tissue was separated and immersed in 10% neutral formaldehyde, and remaining lung tissues were stored at -80°C for subsequent study. The experimental animal methods all conformed to animal ethics standards.

### 2.4. Histopathological analysis

The lung tissue was fixed, dehydrated, transparentized, and embedded in paraffin. After fixation and decalcification, the mice lung tissue was sectioned to 5 μm. H&E staining, Masson staining, and immunohistochemistry was performed separately according to the instructions of the reagent. The section was captured using a Nikon TS2 microscope (Nikon Instech, Tokyo, Japan).

### 2.5. Metabolomics analysis

After the serum samples were defrosted, 100 μL samples were taken and extracted with 300 μL acetonitrile and centrifuged. 330μL supernatant was taken and dried by nitrogen, then centrifuged at 13000 rpm at 4°C for 10min. After redissolving in ultrapure water, 80μL supernatant samples were placed in an inner liner for analysis.

The metabolites in serum were separated by UPLC with an injection volume of 2 μL. The samples were injected into an HSS T3 chromatographic column ((100 * 2.1 mm * 1.7 μm)) at a flow rate of 0.2 mL/min and the column temperature was maintained at 35°C. The mobile phases were 0.1% formic acid (A) and acetonitrile (B). The optimized elution conditions were: 2% B, 0 ~  2 min; 2-35% B, 2-3 min; 35-98% B, 3-28 min; 98% B, 28-32 min; 2% B, 32.1 min, and 2% B at 32.1-34 min to re-equilibrate the column.

The mass spectrometric (MS) scanning was performed using positive and negative mode electrospray ionization (ESI) techniques. And the conditions used were as follows: scanning range was from 400 *m/z* to 1000 *m/z* with a resolution of 70,000; the positive ion source voltage was 4 kV; the negative ion source voltage was 3.5 kV; capillary heating temperature was set to 300°C for both ions; sheath gas pressure and auxiliary gas pressure were 40 psi and 20 psi, respectively (temperature: 350°C, maximum isolation time: 50 ms).

The chromatographic peaks were quantified by compound discover software. SIMCA software (14.1, umetrics, Sweden) was used for principal component analysis (PCA) and orthogonal partial least squares discriminant analysis (OPLS-DA) to visualize the metabolite data results. PCA was used to analyze the expression of differential variation within and between groups. Variable Importance in Projection (VIP) ≥  1 in the OPLS-DA model and the P-values  <  0.05 according to t-test were considered as a potential differential metabolic markers.

### 2.6. Network pharmacology analysis

Network Pharmacology has been used to investigate the compound-target interaction and predict possible mechanisms. Metaboanalyst (https://www.metaboanalyst.ca/), TCMSP (https://tcmspw.com/tcmsp.php), PubChem (https://pubchem.ncbi.nlm.nih.gov/) and SwissTargetPrediction (http://www.swisstargetprediction.ch/) databases were employed to analyze the differential metabolic compounds and predict relevant targets, these metabolite targets were screened to remove duplicates subsequently.

The ingredient and their relevant targets of the drugs in JF were downloaded from the TCMSP database (https://tcmspw.com/tcmsp.php). The drug targets and metabolite targets were imported to find the common targets via EVenn [12] (http://www.ehbio.com/test/venn), then the String database (https://string-db.org) was applied to find the interaction of these common targets. Subsequently, we use Cytoscape 3.9.1 software to filtrate the closely related targets and draw a Protein-Protein Interaction network diagram. To assess the role in signaling pathways of the target proteins we screened, we adopted Gene Ontology (GO) enrichment analysis and Kyoto Encyclopedia of Genes and Genomes (KEGG) metabolic pathway enrichment analysis via the Database for Annotation, Visualization and Integrated Discovery (DAVID) database (https://david.ncifcrf.gov/).

### 2.7. Western blot analysis

The lung tissues were homogenized in RIPA Buffer with 1% protease inhibitor and 2% phosphatase inhibitor. A BCA Protein Assay kit (Beyotime, Shanghai, China) was used to measure the protein concentration. Then proteic samples was separated by sodium dodecyl sulfate polyacrylamide gel electrophoresis, and transferred to a polyvinylidene fluoride (PVDF) membrane. Then the 5% defatted milk was used to block the membrane for 1 h. After that, the membrane was incubated with primary antibody overnight at 4°C, and incubated with secondary antibody for 1 h at room temperature after washed with TBST. Protein expression was normalized to α-Tubulin. The ECL kit (Beyotime, Shanghai, China) was used to visualize the blot, and captured by a chemiluminescence imaging system (Qinxiang Scientific Instruments Co., Ltd., Shanghai, China). The gray value was analyzed by image J software (Image J 1.8.0, National Institutes of Health, Bethesda, Maryland, USA).

### 2.8. Statistical analysis

The data were expressed as the mean ±  standard deviation (SD). The comparisons between two groups were analyzed by Student’s t-test, and the comparisons among multiple groups were evaluated with ANOVA. GraphPad Prism 8.0.2 (La Jolla, CA, USA) were used for statistical analysis of the data. The value of *P*  <  0.05 was considered to indicate a statistically significant difference.

## 3. Results

### 3.1. JF significantly delayed the progression of PF and inhibited the proliferation of fibrous tissue in the PQ-induced mice model

**Fig 1:**
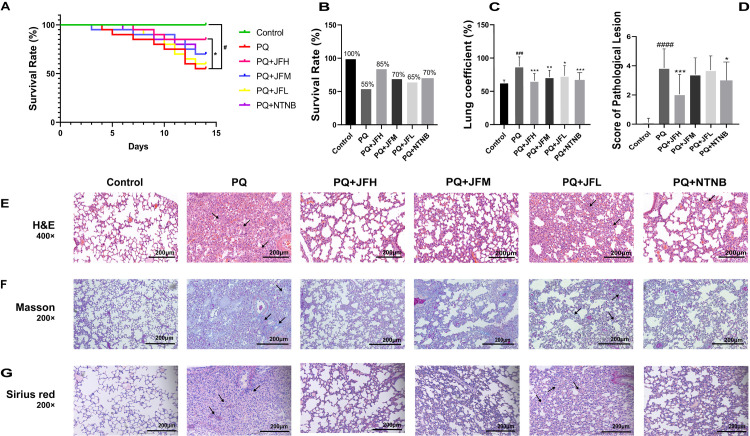
Effect of JF or NTNB on the status of survival and pathological lesions of PF in the PQ-induced mice model. (A) Survival curves of mice of the 6 experimental groups. (B) The survival rates of mice in 6 groups. (C) The lung coefficient of mice. (D) The scores of pathological fibrosis degree of H&E stain. PQ group compare with the normal group, ##P <  0.01 or #P <  0.05; JF group and the NTNB group compare with the PQ group, **P <  0.01 or * P <  0.05. (E) H&E stain of the lung of mice in 6 groups. Scale bar =  200 μm. (F) Masson stain of the lung of mice in 6 groups. Scale bar =  200 μm. (G) Sirius red stain of the lung of mice. Scale bar =  200 μm.

In order to explore the effects of JF on PF, the mice were treated with PQ every day during the 14 days’ experimental period. The survival of mice in the whole experimental period was recorded, and the results of the lung coefficient, survival rate and lung pathological lesions of the mice were shown in Fig 1.

As is shown in Fig 1A–C, the survival rate of PF model mice was significantly reduced (*P* <  0.05), and the lung coefficient increased significantly compared to the control group (*P* <  0.001). However, the survival rates of PF were significantly increased when the PF model mice treated with JFH (1 g/kg), and the survival rates of mice in JFM (0.5 g/kg), JFL (0.25 g/kg) and NTNB (45 mg/kg) groups were increased but not significant. The JF (1, 0.5 and 0.25 g/kg) and NTNB treatment reduced the lung coefficient obviously. These results demonstrated that the intervention of JF obviously inhibited the progression of PF and delayed the death of the mice. The dosage of 1 g/kg JF, which is comparable to 45 mg/kg NTNB, to achieve the same therapeutic effect in PF model mice. Therefore, the dosage 1 g/kg of JF was used in the following mechanism research.

In addition, to explore the inhibited effect of fibrous tissue of JF in the PQ-induced PF mice, the sections of lung tissue were stained by H&E, Masson and Sirius red staining (Fig 1D–G). The results showed that the lung tissue in control group was clear and exhibited no alveolar interval thickening, no obvious exudation in the alveolar cavity, and no inflammation or fibrosis. Compared with the normal group, the lung in PQ group showed abnormal pathological features, which were mainly characterized by the inflammatory substances exuded into the alveolar cavity, local alveolar structure disappeared, and the pulmonary interstitial fibrocyte proliferation was occurred. After treatment with JF and NTNB, the fibroblast proliferation of lung tissue was reduced, the alveolar structure was relatively intact, and the degree of fibrosis was decreased compared with the PQ group. These results indicated that PQ may cause injury in lung and abnormal proliferation of fibroblasts in lung. And the injured alveoli were filled with fibrous tissue, and induced the increased of lung weight and respiratory distress until mice died. JF and NTNB inhibited the abnormal proliferation of fibroblasts.

### 3.2. JF effectively inhibited the fibrosis by down-regulation of TGFβ1/Smad2,3 signaling pathway in PQ-induced PF mice model

**Fig 2:**
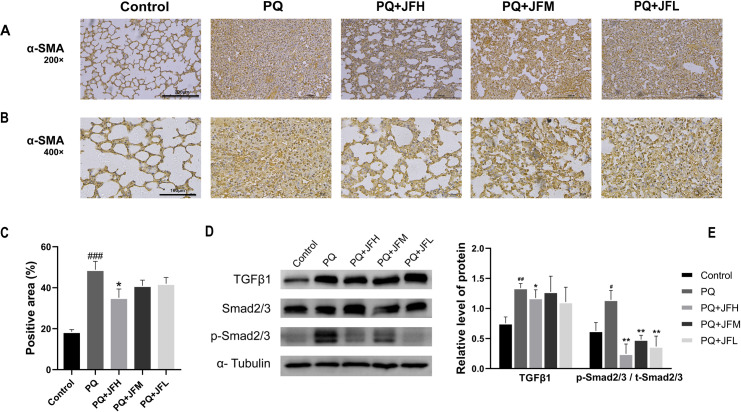
Effect of JF on the expression of α-SMA and TGFβ1/Smad2,3 signaling pathway in PQ-induced PF mice model. (A) Expression of α-SMA measured by immunohistochemistry in 200 × field of microscope. (B) Expression of α-SMA measured by immunohistochemistry in 400 × field of microscope. (C) The proportion of the positive area in expression of α-SMA. (D) Expressions of listed proteins are determined by western blot. (E) The statistically analyzed of the expression of α-SMA and TGFβ1/Smad2,3 signaling pathway. PQ group compare with the normal group, ##*P* <  0.01 or #*P* <  0.05; JF group compare with the PQ group, **P <  0.01 or * *P* <  0.05.

Next, to determine the related effect of JF to the inhibition of tissue fibrosis in PQ-induced PF mice model, the expression of related protein was measured by immunohistochemistry and western blot. The results of α-SMA expression in the lung tissues of was shown in Fig 2A–C. Compared with the control group, the proportion of the positive area of α-SMA was obviously increased, while the distribution of α-SMA expression in the JF intervention groups were significantly reduced.

Moreover, TGFβ1/Smad2,3-mediated fibrosis is closely related to PF [13–16]. The results (Fig 1C and 1D) showed that the expression levels of TGFβ1 (*P*  <  0.01) and p-Smad2, 3 (*P  <  *0.05) were significantly enhanced in PQ group compared with the control group. However, compared with the PQ group, TGFβ1 (*P*  <  0.05), and p-Smad2, 3 (*P  <  *0.01) expressions were significantly decreased in the JFH group; JFM also significantly reduced the levels of p-Smad2, 3 (*P  <  *0.01), but there is no obvious effect on the expression of TGFβ1. The expression of p-Smad2, 3 (*P  <  *0.01) was obviously decreased in the JFL group compared to the PQ group, while no significant effect on the expression of TGFβ1. These results suggested that JF down-regulated the TGFβ1/Smad2,3 signaling pathway and exhibited fibrosis of lung tissue in PQ-induced PF mice model.

### 3.3. JF regulated 16 differential metabolites to achieve the inhibition on PQ-induced PF

**Fig 3:**
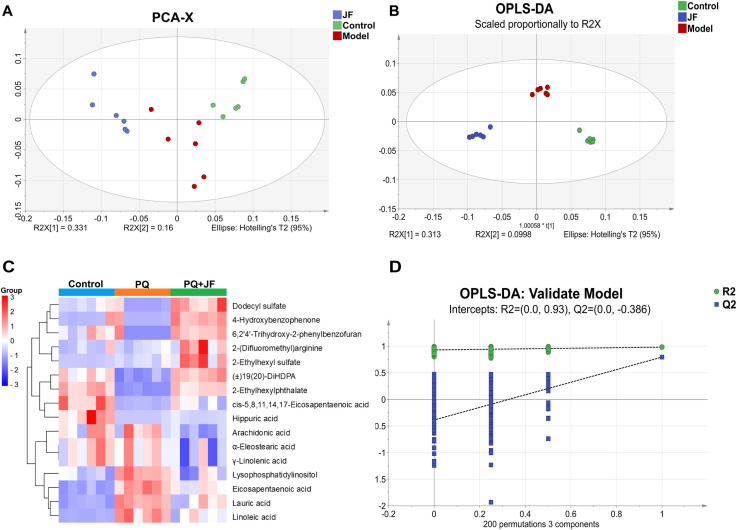
Metabolic spectrum analysis of the mice serum in negative ion mode. (A) The PCA plot of mice serum in negative ion mode. (B) The OPLS-DA plot of samples in negative ion mode. (C) Heatmap of differential metabolites in mice serum in the control, PQ and JF group. (D) The permutation test of the mice serum in the negative ion mode.

Based on the results of pharmacodynamic, liquid chromatography-mass spectrometry (LC-MS) was used to investigate the serum metabolomics and screen the differential metabolites, serum from JFH group mice was used for metabolomics analysis. After preprocessing, 745 and 3468 characteristic peaks were extracted from serum samples in positive and negative ion mode. The principal component analysis (PCA) model (Fig 3A) showed the observably separative trend among the control, PQ and JF groups in the negative ion mode, and there is no significant separation shown among the three groups in the positive ion condition. Therefore, in the subsequent study, we only analyzed the different metabolites detected in the negative ion mode. OPLS-DA was used to identify, characterize and discriminate the differential metabolites in the mice among the Control, PQ and JF groups, and the results (Fig 3B and 3D) showed that the metabolites exhibited significant differences among the control, PQ and JF groups. This suggests that PQ has caused a change in the metabolic profile of the mice, and JF affected the mice metabolism.

The differential metabolites in the serum samples ion modes shown in Fig 3C and Table 1, which screened by FDR adjusted *p-value* < 0.05, and VIP > 1. Level of 16 differential metabolites in the serum were significantly different between the PQ group and the JF group. Compared to that in the PQ group, level of 7 differential metabolites in the serum were upregulated, and level of 9 differential metabolites were downregulated in the JF group. It can be speculated that the protective mechanism of JF to PF was related to the changes of levels of the differential metabolites.

**Table 1:** Identification of significantly different metabolites from control, PQ, and PQ+JF groups.

No.	Name	Formula	VIP	Fold Change (Control/ PQ)	Fold Change (PQ+JF/ PQ)
1	(±)19(20)-DiHDPA	C_22_H_34_O_4_	1.39573	0.77481 ↓*	0.16458 ↓*
2	2-(Difluoromethyl)arginine	C_7_H_14_F_2_N_4_O_2_	1.23169	0.23730 ↓*	0.25441 ↓*
3	2-Ethylhexyl sulfate	C_8_H_18_O_4_S	2.91162	0.02523 ↓*	0.17000 ↓*
4	2-Ethylhexylphthalate	C_16_H_22_O_4_	1.15815	0.74904 ↓*	0.04067 ↓*
5	4-Hydroxybenzophenone	C_13_H_10_O_2_	4.19518	0.21707 ↓*	0.18026 ↓*
6	6,2’4’-Trihydroxy-2-phenylbenzofuran	C_14_H_10_O_4_	1.08496	0.64896 ↓*	0.22147 ↓*
7	Arachidonic acid	C_20_H_32_O_2_	5.43927	1.50183 ↑*	1.50312 ↑*
8	cis-5,8,11,14,17-Eicosapentaenoic acid	C_20_H_30_O_2_	2.65307	2.51053 ↑*	0.17696 ↓*
9	Dodecyl sulfate	C_12_H_26_O_4_S	1.27980	0.37295 ↓*	0.13209 ↓*
10	Eicosapentaenoic acid	C_20_H_30_O_2_	2.96261	0.15570 ↓*	1.79624 ↑*
11	Hippuric acid	C_9_H_9_NO_3_	1.22570	8.50020 ↑*	0.30295 ↓*
12	Lauric acid	C_12_H_24_O_2_	1.72777	0.18821 ↓*	1.86087 ↑*
13	Linoleic acid	C_18_H_32_O_2_	15.07520	0.10468 ↓*	2.35370 ↑*
14	Lysophosphatidylinositol	C_29_H_49_O_12_P	1.69691	0.97122 ↓*	1.97901 ↑*
15	α-Eleostearic acid	C_18_H_30_O_2_	5.91053	1.59196 ↑*	1.55617 ↑*
16	γ-Linolenic acid	C_18_H_30_O_2_	5.91053	1.59200 ↑*	1.56610 ↑*

Note:

* The values were statistically significant (*P  <  *0.05).

↑ The metabolites were up-regulated.

↓ The metabolites were down-regulated.

### 3.4. Integration analysis of network pharmacology and metabolomics of JF on PQ-induced PF mice model

**Fig 4:**
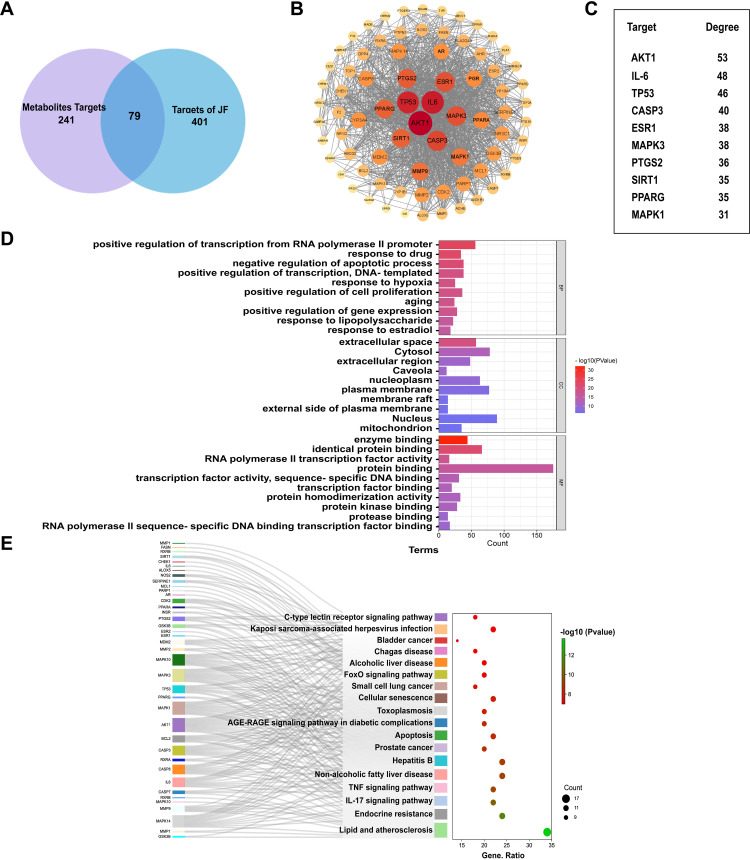
The integration of network pharmacology and metabolomics analysis of PQ-induced PF treatment with JF. (A) The common targets between metabolomics and JF related. (B) The PPI network related to JF and metabolites targets. (C) The top ten targets for Degree Value of PPI network. (D) GO function enrichment analysis of PQ-induced PF treatment with JF. (E) Potential pathways of PQ-induced PF treatment with JF, based on KEGG pathway analysis.

241 relevant targets were acquired for the 16 metabolic differentials screened by the metabolomics via searching the databases. From the TCMSP database, 401 relevant targets were obtained for the action of JF granula, and the Venn diagram showed 79 common targets between the two (Fig 4A). The results were input to the TRING database to obtain the interaction relationships network, the PPI network is established as shown in Fig 4B by Cytoscape software, the size and the color of the nodes which in the network are proportional to the Degree value. There are 79 targets and 630 edges in the PPI network, the average node degree of these targets is 16.2, and the targets with high Degree included Akt1, TP53, IL-6, MAPK3, Caspase-3 and so on (Fig 4C), which indicating that the effect of JF on PQ-induced metabolism in mice with PF may be related to apoptosis and inflammation.

DAVID database was employed to analyze the Gene Ontology and KEGG pathway analysis (Fig 4D and 4E), GO enrichment analysis showed that these targets were involved in apoptotic process, including positive or negative regulation of apoptotic process, and also involved in positive or negative regulation of transcription of RNA polymerase II promoter, and phosphorylation of protease serine. KEGG pathways enrichment showed that the metabolism of JF may be involved in the regulation include IL-17 signaling pathway, TNF signaling pathway, Apoptosis, AGE-RAGE signaling pathway for diabetic complications and so on.

### 3.5. JF regulated the apoptosis and the PI3K/AKT signaling pathway in PQ-induced PF mice model

**Fig 5:**
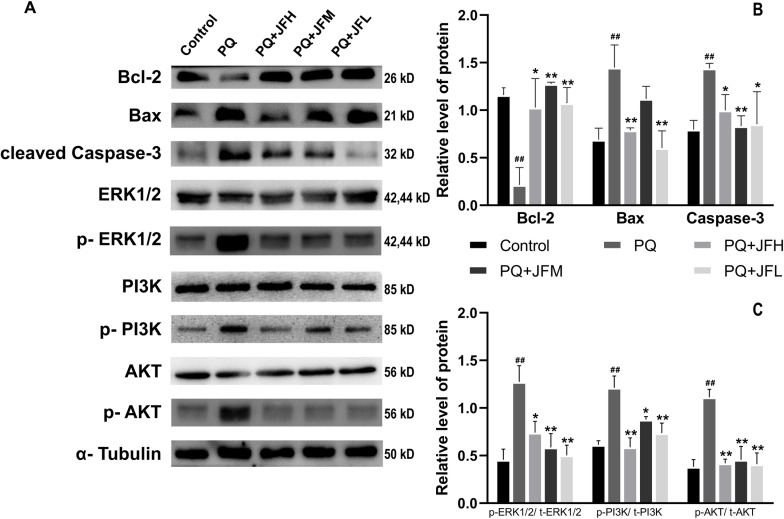
The expression of Bcl-2, Bax, Caspase-3, p-ERK1/2, p-PI3k and p-Akt in pulmonary tissues recorded by western blot. (A) The expression of Bcl-2, Bax, Caspase-3, ERK1/2, p-ERK1/2, PI3k, p-PI3k, Akt and p-Akt in pulmonary tissues recorded by western blot. (B) Quantitative result of Bcl-2, Bax, Caspase-3. (C) Quantitative result of p-ERK1/2, p-PI3k and p-Akt. Data reported in the figures are mean ±  SD, PQ group compare with the normal group, ##*P* <  0.01 or #*P* <  0.05; JF group compare with the PQ group, **P <  0.01 or * *P* <  0.05.

To determine whether apoptosis was involved in the anti-PF effects of JF, the expression of apoptosis-related proteins Bcl-2, Bax, Caspase-3 were detected. The results (Fig 5A and 5B) showed that the expression of Bax (*P*  <  0.01) and Caspase-3 (*P*  <  0.01) was significantly increased, and Bcl-2 was reduced obviously (*P  <  *0.01) in the PQ-treated group compared with the control group. And compared with the PQ group, the expression of Bax (*P*  <  0.01) and Caspase-3 (*P  <  *0.05) were significantly decreased intervened by JFH, while Bcl-2 was increased (*P  <  *0.05) visibly in the JFH group; JFM decreased the level of Caspase-3 (*P  <  *0.01) and elevated Bcl-2 (*P  <  *0.01) levels distinctly, while effected inconspicuously on the expression of Bax. The expression of Bcl-2 (*P*  <  0.01) was conspicuously enhanced, Bax (*P*  <  0.01) and Caspase-3 (*P  <  *0.05) were significantly decreased in the JFL group compared to the PQ group.

In addition, to further verify the results of metabolomics and network pharmacology, we detected the expression of ERK1/2 (MAPK3), p-ERK1/2, PI3k, p-PI3k, Akt, p-Akt. The results of western blot analysis (Fig 5A and 5C) revealed that the expression levels of p-ERK1/2, p-PI3k, and p-Akt was distinctly higher in the PQ group than in the control group (*P*  <  0.01), while JF treatment, included H, M, L doses, almost totally abolished paraquat-induced upregulation of p-ERK1/2, p-PI3k, and p-Akt expressions (*P  <  *0.01 or *P  <  *0.05). The results show that JF can inhibit the development of paraquat-induced PF by inhibit the apoptosis, and also corroborates the results of metabolomics and network pharmacology.

## 4. Discussion

JF is an ancient herbal remedy, which is the combination of multiple herbs with complex composition. As a clinical drug, JF is used for anemofrigid cold, headache, nasal congestion, clear snot, cough with white phlegm, etc. It is a challenge to understand and interpret the effects and mechanisms of PQ-induced PF treated with JF. This study explained the inhibitory effects and mechanisms of JF on PQ-induced PF by the integrate of pharmacodynamics, metabolomics and network pharmacology. The direction of JF shows the daily dosage for adults is 6.9 g/kg/day, so that the equivalent dosage of JF for mice is 1.05 g/kg/day, so the mice in administration groups (H, M, L) received JF at 1 g/kg, 0.5 g/kg and 0.25 g/kg daily, respectively.PQ, a toxic bipyridilium pesticide, is mainly accumulated in lung after entering in body. It causes alveolar epithelial cell rupture, edema, interstitial changes and inflammatory cell infiltration [17], increased fibroblast proliferation and collagen deposition eventually lead to PF and even death [18]. Therefore, PQ was used to play an inducer role to establish the PF mice model in this study.

It is evident from the survival curve that PQ caused a significant decrease in the survival rate of mice, which showed obvious signs of intoxication, and the intervention of JF increased the mice survival rate with increasing dose. Analogously, the results of the lung coefficient showed PQ made the lung coefficient of mice dramatically higher, while the lung coefficient of mice in all dose groups of JF was significantly reduced compared with the PQ group, and the JFH group was especially significant. The positive control of NTNB had the similar effects to JF. The results indicated that the clinical dose of JF had an inhibitory effect to the toxic, and delayed the death of mice caused by PQ.

The results of H&E, Masson and Sirius Red staining in this study showed that JF treatment significantly reduced the degree of alveolitis and fibrosis. Preliminarily, it was confirmed that JF attenuated the pathological damage of lung tissue in PQ-intoxicated mice. Myofibroblasts are the main cells that secrete the collagen, and the persistent abnormalities of myofibroblasts is one of the important signs in pulmonary fibrotic disease. The results showed the abnormalities myofibroblasts increased in PQ group, the model of PF induced by PQ was established, while after JF treatment the abnormalities of myofibroblasts reduced, and destruction of alveolar structure was obviously alleviated. From the results of immunohistochemistry, the proportion of positivity area of α-SMA was markedly increased in the PQ group, but decreased in JF groups. α-SMA is one of the phenotypic markers of myofibroblasts. It indicated the decreased of myofibroblasts on PQ-induced mice PF with the JF intervention. In addition, the activation of fibroblasts is the central mechanism of PF, and transforming growth factor β1 (TGF-β1) plays a vital role, and TGF-β1 directly activates the Smad2, 3 signaling, whose pathological activation was correlated with fibrosis development [19,20]. The results of western blot showed that PQ activate the TGF-β1/ Smad2, 3 signs, and JF down-regulated the expression of them and against the PQ poisonousness to inhibit the PF. These results demonstrated that JF can achieve the inhibit effect by reduced the collagen secreted and down-regulated the TGF-β1/ Smad2, 3 pathway.

Metabolomics was used to examine the differences in metabolism between groups of mice serum to reveal the related diseases of the treatment with drugs and their mechanisms, which is a common method for TCM today [21,22]. In this study, metabolomics was employed to explore the mechanism of JF for the treatment of PQ-induced PF, find the differential compounds, and their potential targets. The significant differential metabolites were obtained and screened included Arachidonic acid (AA), Linoleic acid (LA), Eicosapentaenoic acid (EA), Lysophosphatidylinositol (LPI), etc. The regulation effect of JF on metabolism of PQ-induced PF might be related to the biosynthesis of unsaturated fatty acids.

Unsaturated fatty acids, a type of fatty acid that constitutes body fat, are a major component of cell membranes and are essential for maintaining the relative fluidity of cell membranes and normal cellular physiological functions. Recent studies have found that abnormal changes in unsaturated fatty acids are closely related to PF [23–26]. For instance, the disorders of fatty acid metabolism and imbalance in the ratio of monounsaturated fatty acids promote the development of PF [27]; the stearic acid could exert antifibrotic activity by modulating pro-fibrotic signaling activity [28]. LPI is one of the significant differential metabolites screened between JF and PQ groups, it participates in the metabolism pathway of Glycerophospholipids (GPLs). GPLs are the main components of biological membranes and play a crucial role in the function of cells [29]. The GPLs metabolism pathway produces various metabolites such as phosphatidylcholine (PC), LPI, lysophosphatidylethanolamine (LysoPE), phosphatidylethanolamine (PE), and lysophosphatidylcholine (LysoPC) [30], which are the crucial differential metabolites in the progression of PF. LysoPC induces the PF by mediating vascular leakage and fibroblast migration and proliferation [31]. And a research of blood levels of LPC and LPC were found to be significantly elevated in patients with idiopathic pulmonary fibrosis [32]. PC and PE are the key lipids of pulmonary surfactant, which were reported to be significantly increased in PF [33,34]. The abnormalities of these metabolites in GPLs metabolism pathway lead the dysregulation of lipid metabolism, which is characteristic of many respiratory diseases. It can be inferred that the inhibitory effect of JF on PQ-induced PF may be related to the regulation of abnormal lipid metabolism of GPLs metabolism pathway. This result provides a new perspective for further research in the future.

Arachidonic acid (AA) is a polyunsaturated fatty acid and intercellular communication mediator, playing multiple roles in disease pathogenesis, such as anti-inflammatory, antifibrogenic and antiapoptotic [35]. According to the Metabolomics findings, AA is one of crucial metabolic pathways for JF to the treatment of PQ-induced PF. AA is primarily metabolized via the lipoxygenase (LOX) and cyclooxygenase (COX) pathways, which are crucial for the production of inflammatory mediators. It has been reported that when inflammation occurs, AA in the cell membrane produces a range of physiologically active mediators, such as leukotrienes, prostaglandins and lipoxygenins in the presence of LOX and COX. These mediators trigger an inflammatory response and promote the development of fibrosis [25,36,37]. These derivatives form the foundation of various epidemic disease pathologies, ultimately leading to tissue fibrosis, including in the kidney [38,39], liver [40,41] and lung [42,43]. However, some previous studies found that AA metabolized through the cytochrome P450 pathway, and produced active mediators including prostaglandin E2, lipoxin A4, Epoxyeicosatrienoic acids and prostacyclin, which have shown the antifibrotic effects on fibrosis models [44,45]. The specific regulatory effect of JF on the AA metabolic pathway remains unknown, requires the further exploration and research.

The JF-related targets and metabolic-related targets were integrated by network pharmacology, 79 common targets were found and established the PPI networks. The key targets including Akt, TP53, IL-6, Caspase-3, MAPK3, etc were identified, which indicated the anti-apoptotic and anti-inflammatory properties of JF in inhibiting the PF. GO analysis of these targets revealed that the inhibitory effects of JF on PF involved 308 biological processes, 121 cellular components and 84 molecular functions. As shown by the results, apoptosis is a crucial biological process in the treatment of JF of PQ-induced PF. Further, KEGG analysis indicated that the inhibitory effect may be related to lipid and atherosclerosis pathway, TNF, apoptosis, PI3k-Akt and IL-17 signaling pathways. Subsequently, these targets were verified and found that JF could inhibit the expression of Bax and Cleaved-Caspase-3, while increase the expression of Bcl-2, thus exerting an anti-apoptotic effect, consistent with the predicted results.

Akt is the main downstream regulatory protein of PI3k, also known as protein kinase B, which plays a crucial role in cell survival and proliferation [46]. PI3k/Akt is a classical signaling pathway, which the activation is a common mechanism for intracellular signaling by lots of growth factors and inflammatory mediators [47–49]. PI3k is a complex family of dimeric proteins composed of catalytic and regulatory subunits, and they have dual activities as lipid kinases and protein kinases, which act to catalyze the phosphorylation of LysoPC [50], thereby activating downstream regulatory proteins, this process coincides with the results of metabolomics in this study. It suggested that the regulatory effect of JF on PQ-induced PF may be closely related to the regulation of the PI3k/Akt pathway. It confirmed that PQ allows the activation of PI3k/Akt pathway with elevated expression, suggesting an increase in fibroblast proliferation, while JF regulated this pathway and inhibit the abnormal proliferation of fibroblasts.

## 5. Conclusion

In summary, effect and mechanism of JF treatment for PQ-induced PF were preliminary interpreted by integrative analysis of pharmacodynamics, network pharmacology and metabolomics. JF can regulate the TGFβ1/Smad2,3 and PI3K/AKT pathways to display the favorable effect of inhibiting the progression of PF and delay the survival of mice. Arachidonic acid and Lysophosphatidylinositol were possibly the key metabolic pathway of JF in PQ-induced JF mice model. This study demonstrates the protective effect of JF on PF, which is the development of new indications for JF and provides new alternative therapeutic drugs for the treatment of PF.
